# Supplementary material for: Translational control of ERK signaling through miRNA/4EHP-directed silencing
Source: eLife. 2018 Feb 7;7:e35034. doi: 10.7554/eLife.35034 (PMC5819943; doi:10.7554/eLife.35034)
Supplement: Supplementary file 2. — Highlighted sequence represent the translation stop codon. [file elife-35034-supp2.docx]

**Supplementary file 2.** Dusp6 3´ UTR isolated from U251 human glioblastoma cell line. Highlighted sequence represent the translation stop codon.

TGAAAGACCCCACACCCCTCCTTGCTGGAATGTGTCTGGCCCTTCAGCAGTTTCTCTTGG CAGCATCAGCTGGGCTGCTTTCTTTGTGTGTGGCCCCAGGTGTCAAAATGACACCAGCTG TCTGTACTAGACAAGGTTACCAAGTGCGGAATTGGTTAATACTAACAGAGAGATTTGCTC CATTCTCTTTGGAATAACAGGACATGCTGTATAGATACAGGCAGTAGGTTTGCTCTGTAC CCATGTGTACAGCCTACCCATGCAGGGACTGGGATTCGAGGACTTCCAGGCGCATAGGGT AGAACCAAATGATAGGGTAGGAGCATGTGTTCTTTAGGGCCTTGTAAGGCTGTTTCCTTT TGCATCTGGAACTGACTATATAATTGTCTTCAATGAAGACTAATTCAATTTTGCATATAG AGGAGCCAAAGAGAGATTTCAGCTCTGTATTTGTGGTATCAGTTTGGAAAAAAAAATCTG ATACTCCATTTGATTATTGTAAATATTTGATCTTGAATCACTTGACAGTGTTTGTTTGAA TTGTGTTTGTTTTTTCCTTTGATGGGCTTAAAAGAAATTATCCAAAGGGAGAAAGAGCAG TATGCCACTTCTTAAAACAGAACAAAACAAAAAAAGAAAATTGTGCTCTTTTCTAATCCA AAGGGTATATTTGCAGCATGCTTGACTTTACCAATTCTGATGACATCTTTACGGACACTA TTATCACTAAGACCTTGTTATGGCGAAGTCTTTAGTCTTTTTCATGTATTTTCCTCATGA TTTTTTCTCTTTATGTAGTTTGACTATGCCTTACCTTTGTAAATATTTTTGCTTGTGTTG TCGCAAAGGGGATAATCTGGGAAAGACACCAAATCATGGGCTCACTTTAAAAAAAGAAAG AATAAAAAAACCTTCAGCTGTGCTAAACAGTATATTACCTCTGTATAAAATTCTTCAGGG AGTGTCACCTCAAATGCAATACTTTGGGTTGGTTTCTTTCCTTTAAAAAAATTTGTATAA AACTGGAAGTGTGTGTGTGTGAGCATGGGTACCCATTTGATAAGAGAAATGCATTTGATT GTGAAGAAGGGAGAGTTAAATTCTCCATTATGTTCGTGGTGTAAAGTTTAGAGCTGGAAT TTATTATAAGAATGTAAAACCTTAAATTATTAATAAATAACTATTTTGGCTATTG
